# The effect of glycyrrhizin acid on *Bax* and *Bcl2* expression in hepatotoxicity induced by Titanium dioxide nanoparticles in rats 

**Published:** 2020

**Authors:** Mahmoud Orazizadeh, Layasadat Khorsandi, Esrafil Mansouri, Fereshtesadat Fakhredini

**Affiliations:** 1 *Cell & Molecular Research Center, Faculty of Medicine, Ahvaz Jundishapur University of Medical Sciences, Ahvaz, Iran*; 2 *Department of Anatomical Sciences, Faculty of Medicine, Ahvaz Jundishapur University of Medical Sciences, Ahvaz, Iran *

**Keywords:** NTiO2, Immunohistochemistry, TUNEL, Apoptosis, Histopathological

## Abstract

**Aim:**

This research studied the effects of glycyrrhizic acid (GA) on apoptosis induced with by titanium dioxide (NTiO2) in the liver of rats.

**Background:**

It is widely accepted that the contamination resulting from nanoparticles (NPs) is an emerging dangerous issue. Metal oxide nanoparticles have high environmental stability and cause toxicity in the food chain. Thus, the present study investigated the anti-apoptotic effects of glycyrrhizic acid (GA) on the hepatotoxicity generated by titanium dioxide (NTiO2) NPs in the liver tissue.

**Methods:**

Thirty-two male Wistar rats were randomly divided into four groups. NTiO2-treated rats were given 300 mg / kg of NTiO2 solution via gavage for 14 days; GA-treated were administered 100 mg/kg GA for 14 days; protection group was pre-treated with GA before NTiO2 administration for 7 days. Then, apoptotic index was evaluated through immunolocalization of Bax and Bcl-2 and TUNEL assay.

**Results:**

we found that HSCORE of Bax expression and apoptotic index experienced a significant increase with NTiO2 (P <0.001), while Bcl-2 expression significantly diminished in NTiO2-treated rats (P <0.001). The results revealed that the increased Bax expression and apoptotic index were reversed by GA and enhanced the activities of Bcl2.

**Conclusion:**

The results revealed that GA effectively attenuated apoptosis against NTiO2 in rats.

## Introduction

 Researchers agree that the liver is a target organ for adverse impacts of xenobiotics. The metabolic functions and strategic location of the liver in close proximity to the gastrointestinal tract and bloodstream have made it highly susceptible to chemical damage, including the detrimental effects of various drugs and chemical pollutants. It is also targeted for various toxins and susceptible to diseases such as liver toxicity (1). Some researchers have found that administrating NPs to the rodents causes accumulation of NPs in diverse tissues such as the brain, liver, and spleen (2, 3).

Amongst the numerous metal nano-materials, it has been demonstrated that NTiO2 is utilized in a wide range of consumers' products such as electronics, sunscreen, paints, cosmetics, clothing, and surface coating (4, 5). Further, NTiO2 is extensively utilized in synthesizing toothpaste, food colorants, and nutritional supplements. The main pathways of exposure to NTiO2, causing toxic communication, are inhalation and skin exposure. Although most exposure to NTiO2 is through epidermal contact, numerous studies have reported that NTiO2 does not cause skin infections (6, 7). Since the respiratory tract is one of the key exposure ways, most investigations on the NTiO2 have emphasized pulmonary toxicity. For example, Oberdorseter et al. (2002) showed that the NPs were translocated and accumulated in the liver after the inhalation of carbon particles (8). In addition, Fabian et al. (2008) examined Tissue distribution and toxicity of intravenously administered titanium dioxide nanoparticles in rats. Male Wistar rats were treated with single intravenous injections of a suspension of TiO2 in the serum (5 mg/kg body weight), and the tissue content of TiO2 was determined 1, 14, and 28 days later. TiO2 levels were retained in the liver for 28 days (9). Sugibayashiet et al. (2008) achieved the same outcomes (10). 

It has been shown that NTiO2 has apoptotic effects on several cell lines such as brain cells (11), osteoblasts (12), and induction of necrosis in fibroblasts (13). TiO2 NPs can penetrate hepatocytes of liver, which is an active organ for detoxification. Liu et al. (2009) (14) observed a considerable reduction of the body weight of the rat treated with nano anatase TiO2 and an increase in the weight of the liver. Ma et al. (2009) (15) found that TiO2 NPs damage the liver function and induce an oxidative stress attack causing hepatotoxicity. Li et al. (2012) (16) reported the levels of gene expression through TiO2 NPs associated with reactive oxygen species (ROS) and cytochrome p450 (CYP1A). In addition, CYP1A activation causes creation of free radicals and ROS, which initiate lipid peroxidation and protein oxidation and cause damages to the hepatocellular membranes. This is followed by releasing inflammatory mediators from the activated hepatic macrophages that cause hepatic necrosis.

Herbal medicines produced from herb extracts have been applied for treating a wide range of clinical illnesses. Note that the defensive impacts of natural anti-oxidants against chemical-induced toxicity have been studied extensively (17,18). Liquorice has been used in food and as medicine for several centuries. Further, licorice root, which is called 'sweet root', consists of compounds almost 50 times sweeter compared to sugar (18).

A study found that glycyrrhizin acid** (**GA) is one of the natural constituents of liquorice separated from the dried root of Glycyrrhizaglabra. It has also been observed that GA salts have extensive applications as aromatizes and sweeteners in sweets, drugs, beverages, chewing-gum, chewing tobacco, and toothpaste (19). Meanwhile, some researchers have demonstrated several pharmacologic impacts of GA such as anti-inflammatory, neuro-protection, antiviral, antitumor, antioxidant (20–26) as well as hepatoprotective activity (26–31). Since the number of patients with liver failure has increased in recent years and liver disorders are the leading to mortality in today's world, we therefore evaluated the therapeutic effect of GA on NtiO2-induced apoptosis in hepatocytes and examined the structure of the liver tissue (32-35). 

## Methods

**Animals**

The study participants consisted of 32 adult male Wistar rats (age: ranging between 8 and 10 weeks with a weight of 180 to 220 g). Ahvaz Jundishapur University of Medical Sciences, Experimental Research Center was selected to prepare the animals. The ethics committee (CMRC-81) of Jundishapur University gave the approval of the current research. According to the research design, the rats were kept under standard laboratory conditions (a 12 h dark & 12 h light cycle, relative moisture of 50±5% & temperature of 22 ± 3 °C) for a minimum of one week prior to testing. The above conditions were conserved until the test was ended.

**Experimental design**

Based on the research protocol, the animals were classified into four groups of eight animals randomly:

Group 1: Control group, received saline by gavage for 21 days.

Group 2: GA group, received 100 mg/kg glycyrrhizic acid (GA) by gavage for 21 days.

Group 3: NTiO2-intoxicated group, 0.2 ml saline was administered for 7 days, and then 300 mg/kg NTiO2 was given for 14 days.

Group 4: Protection group, 100 mg/kg GA was administered for 7 days, and then GA (100 mg/kg) plus NTiO2 (300 mg/kg) was given for 14 days.

According to the research design, the dosages of NTiO2 (Sigma) were chosen based on earlier investigations revealing considerable poisonousness in animals (36). When the NTiO2 NPs were characterized (outcomes not presented), we procured the stock solution (2 mg/ml) in distilled water. Then, we distributed it for ten minutes by a sonicator. Afterwards, the stock solution of NTiO2 was placed at temperature of 4 °C and utilized over 1 week. Just before the experiment, the stock solution was diluted using distillate water and dispersed through ultrasonicating (Solid State/Ultrasonic FS-14: Fisher Scientific) for fifteen minutes in order to avoid accumulation. In order to ensure the non-accumulation of NTiO2 prior to administration, oral gavage was performed over <20 minutes after preparation. Next, after 20 min past the procurement, the size of the NTiO2 particle was measured using atomic force microscopy (AFM). We dissolved 4200 mg of GA (sigma) in 90 cc of saline. The GA dosage was chosen according to the outcomes of earlier research (37) and our pilot study. In brief, diverse dosages of GA (10, 50, 100, 200 mg/kg) were studied for 2 weeks prior to the test to obtain the most acceptable dosage for protecting the liver with four rates utilized to study all dosages. Biochemical tests including ALT and AST were evaluated after 14 days. ALT and AST were significantly reduced at the doses of 200 and 100 mg/kg (Fig. 2). Thus, we used GA at the dose of 100 mg/kg in this experiment. Then, 1 day following the final administration, animals were killed via anesthesia and their livers were removed. Afterwards, formalin 10% was used to fix them for immunohistochemistry analysis. 

**Biochemical tests**

The blood sample were collected in a heparinized centrifuge tube and centrifuged. The plasma enzyme levels including plasma alanine aminotransferase (ALT) and aspartate aminotransferase (AST) were determined spectrophotometrically from plasma samples using commercially available kits (Sigma).

**Immunohistochemistry assay**

The current research carried out deparaffinization and rehydration for Bax and Bcl-2 immunoreactivity using xylene and alcohol. Then, distilled water was used to wash them, and citrate buffer (10 mmol/L) was employed for pretreatment of the sections into a microwave oven for 20 min. Then, 1% H2O2 for 20 min and a protein block solution (Dako, Carpinteria, CA) were used to treat these sections for 10 min. In order to specify the expression of Bcl-2 and Bax proteins, the rat monoclonal antibody against Bcl2 (sc-7382, Santa Cruz) and Bax (sc-20067, Santa Cruz) in 1:50 was utilized for 90 minutes. Accordingly, 1:300 peroxidase-conjugated streptavidin (Dako A/S) and 1:200 bio-tinylated anti-rat antibodies (Dako A/S; Copenhagen: Denmark) were used to incubate the sections. Lastly, 3, 3’diaminobenzidine (DAB) tetra-hydrochloride (Sigma; St Louis: MO) was utilized for establishing the peroxidase reaction and hematoxylin for counterstaining. Immunostaining intensity was assessed by a semiquantitative score called HSCORE technique. This method was computed for all sections via applying the subsequent algorithm (38); that is, HSCORE=ΣPi (i+1) where i refers to the staining intensities (0: no staining, 1= weak, 2= moderate, 3=strong). Pi refers to the percentages relating to the stained cells of each individual intensity (0 to 100%). 

**TUNEL assay**

In-Situ Cell Death Detection Kit, POD (Boehringer Mannheim GmbH; Mannheim; Germany) was utilized to detect the particles at a single cell level according to the labeling directions of the DNA strand breaks. Then, the paraffin sections were dewaxed. Afterwards, rehydration of the sections was performed using common techniques. Next, protease was added and incubation was performed with 5% of suitable normal serum (for 30 minutes at 37 °C). Then, phosphate buffered saline was used to rinse the slides. After permeation of sections for two minutes on ice, TUNEL reaction mix was used to incubate them for 60 minutes at 37 °C. After adding Antifluorescein-AP, they were incubated for 30 min again at 37 °C. Accordingly, PBS was used to wash the sections, and incubation was done for 20 minutes using the substrate. Then, light microscopy was employed to analyze them. Afterwards, apoptotic index (AI) was computed via dividing the number of TUNEL-positive germ cell in a randomly focused somniferous tubule by the total numbers of the germ cells in that tubule. Next, the obtained outcome was multiplied by 100. Finally, AIs corresponding to the ten randomly chosen tubules for all spermatogenic steps were assessed, and thus the average AI of all cases was computed (39).

**Statistical analyses**

According to the research design, one-way ANOVA and post-hoc LSD test were used to analyze the data. The results are expressed as the mean±SD. In addition, p greater than 0.05 was regarded statistically significant.

## Results

**Nanoparticle characterization**

**Figure 1:**
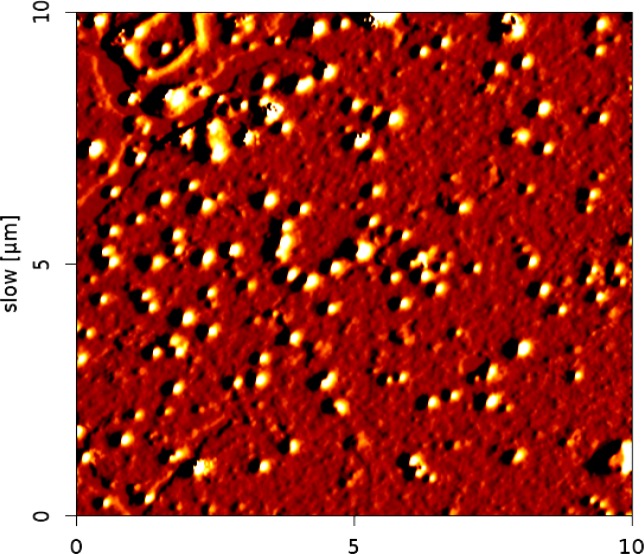
AFM image of NTiO2 showed distinct spherical particles in the size range between 50 and 100 nm

As seen, AFM showed the dimension and morphology of the prepared particles. It was indicated that complexes had spherical shapes with an average dimension less than 100 nm (Figure 1).

**Biochemical tests**

**Figure 2:**
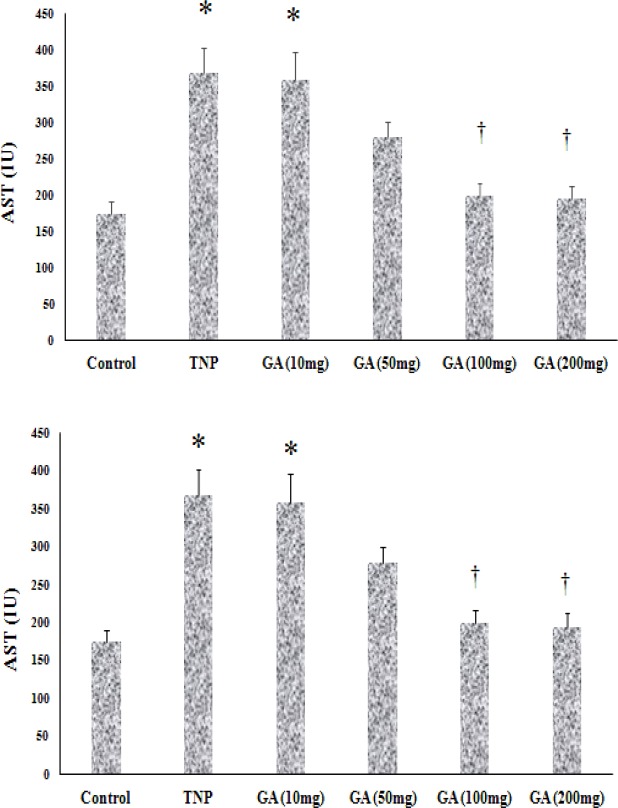
Pilot results for an appropriate dose of GA. The values are expressed as mean ± SD. (* P <0.05), (†p < 0.05). * and † symbols indicate comparison to control and NTiO2-intoxicated groups respectively

ALT and AST were evaluated after 14 days. ALT and AST were significantly reduced at the doses of 200 and 100 mg/kg (Fig. 2). Thus, we used GA at the dose of 100 mg/kg in this experiment (Figure 2).

**IHC assessments**

**Figure 3:**
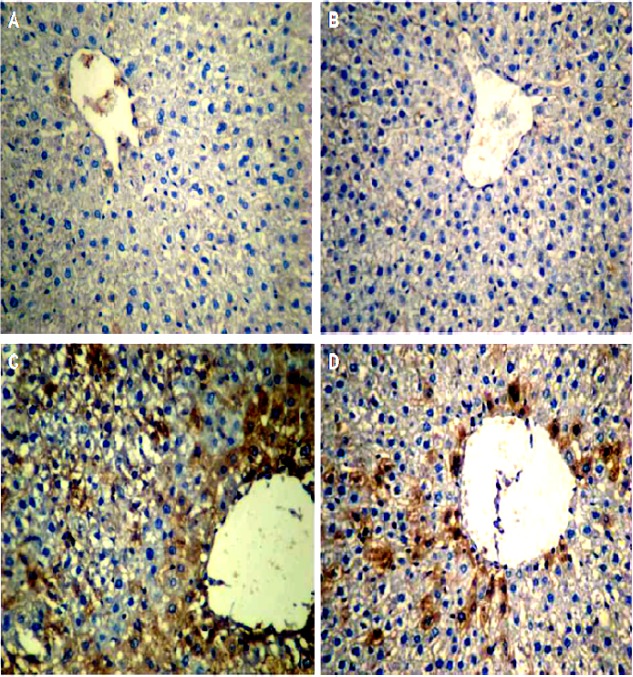
The optical microscope image of the rat liver in the control group (A), GA (B), NTiO2(C) and GA + NTiO2 (D) group by IHC staining of Bax protein. In the control group and GA, no color is observed, but in the NTiO2 group in the cytoplasm, hepatocytes have expressed the Bax (brown) protein. In the GA + NTiO2 group, this protein is also less pronounced than in the NTiO2 group (250 ×).

A weak IHC reaction was observed in some of the lobules of the control group (Figure 3). The expression pattern of Bax in GA-exposed liver was similar to that of the control group. Also, the results of HSCORE showed no significant difference between them. Further, in most lobules of the NTiO2 group, Bax was expressed in hepatocytes, especially around the central vein with different color intensities. In addition, HSCORE experienced a significant rise in comparison with the controls and GA groups (P<0.001). Finally, the color intensity and the number of the stained cells in the GA + NTiO2 group were lower than those of the 

**Figure 4:**
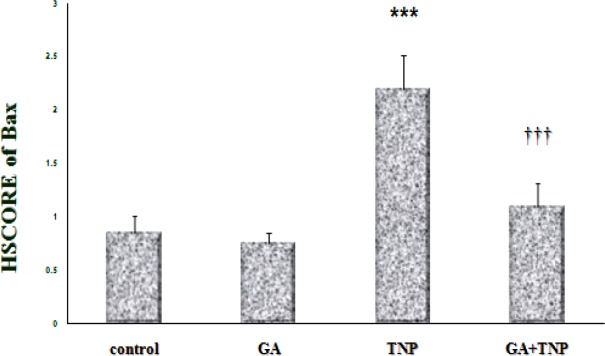
HSCORE assessments of Bax protein in the control groups, GA, NTiO2 and treatment (GA + NTiO2). The values are expressed as mean ± SD. (*** P <0.001), (††† p < 0.001). * And † symbols indicate comparison to control and NTiO2-intoxicated groups respectively

NTiO2 group. Meanwhile, the results of HSCORE showed that Bax expression was significantly reduced in this group in comparison with the rats in NTiO2 group (P<0.001) (Figure 4).

**Figure 5:**
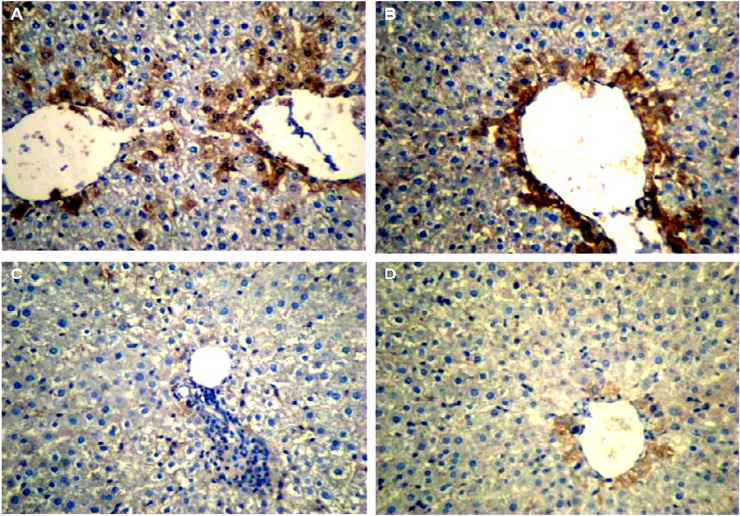
The optical microscope image of the rat liver in the control group (A), GA (B), NTiO2 (C) and GA + NTiO2 (D) group by immunohistochemical staining of Bcl-2 protein. In the control group and GA, the expression of Bcl-2 protein (brown color) is observed, but in the NTiO2 group, the expression of Bcl-2 protein is not observed in hepatocyte cytoplasm. In the GA + NTiO2 group, this protein is more pronounced than in the NTiO2 group (250 ×).

**Figure 6:**
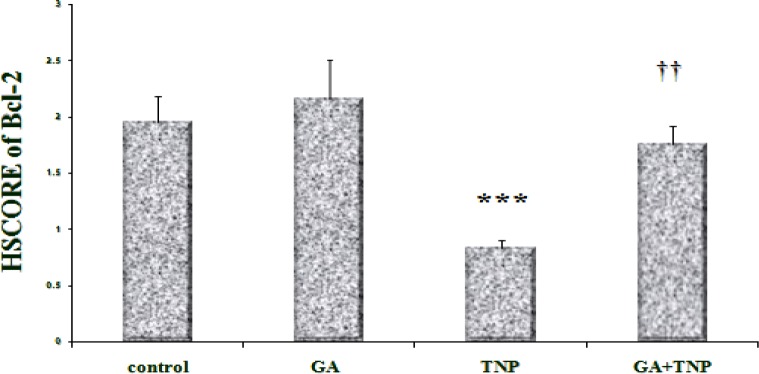
Comparison of Bcl-2 protein expression in control groups, GA, NTiO2and treatment (GA + NTiO2). The values are expressed as mean ± SD (†† p< 0.01), (*** p < 0.001). * And † symbols indicate comparison to control and NTiO2-intoxicated groups respectively

According to the results, in the control group (Fig. 5), Bcl-2 was expressed around the central vein. In the GA group (Figure 5), the expression pattern of Bcl-2 was similar to that of the control group and the results of HSCORE showed no significant difference between them (Figure 6). However, in the NTiO2 group (Figure 5), the number of the stained cells and color intensity were lower than those of the control and GA groups. In addition, the results of HSCORE showed a significant fall compared to the GA and control groups (Figure 6). In the GA + NTiO2 group (Figure 5), the color intensity and the number of the stained cells considerably increased compared to the NTiO2 group, and the results of HSCORE also showed a significant growth (Figure 6).

**Figure 7:**
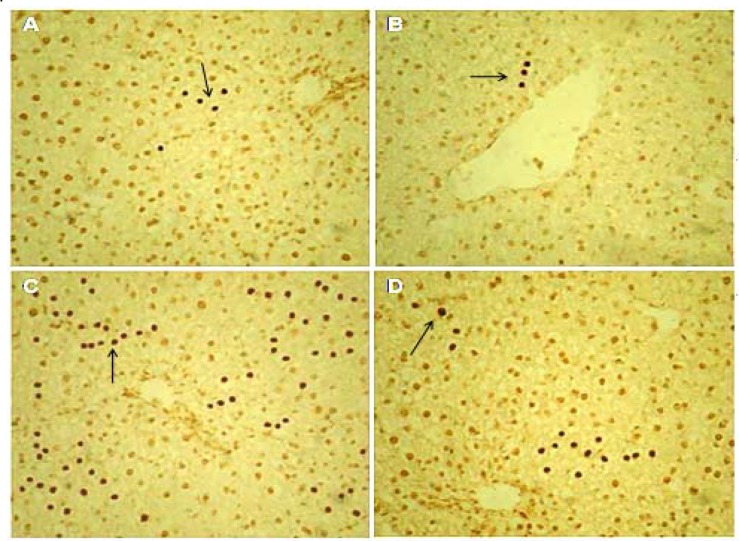
Light microscopy of cross-sections of TUNEL stained liver from control and experimental groups. (A) Control group; (B) GA group; (C) NTiO2-intoxicated group; (D) NTiO2 + GA group. Arrows indicate TUNEL-positive reaction. Magnification: 250×

**Figure 8.**
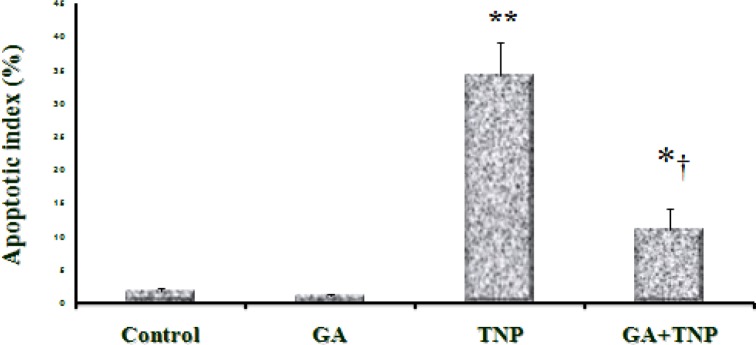
Apoptotic index of control and experimental groups. Values expressed as mean ± SD for 8 mice. **p < 0.01, ***p < 0.001, †† p < 0.01, ††† p < 0.001; * and † symbols respectively indicate comparison to control and NTiO2- intoxicated group

**TUNEL assessments**

Spontaneous apoptosis was mostly found in several normal liver hepatocytes associated with the controls (Fig. 7). Based on the results, apoptotic index in the GA group was partially lower compared to the controls (p > 0.05). Further, apoptosis was significantly enhanced in all lobules of the NTiO2 group compared to the controls (p <0.001). On the other hand, the apoptotic index in the NTiO2+GA group considerably diminished in comparison with the rats treated with NTiO2 (p <0.05) (Figure 7 and 8).

## Discussion

A previous report showed that high concentrations of iron oxide nanoparticles (150 μg / kg) significantly increased the serum levels of the liver enzymes (40). Also, this study demonstrated that GA effectively attenuated apoptosis and histological changes in the hepatotoxicity induced via NTio2. IHC evaluations revealed that the expression of Bax increased in the hepatocytes of the rats receiving NTiO2 significantly; however, the expression of Bcl-2 in this group significantly decreased.

In addition, some previous studies found that NTiO2 in the liver led to induction of expression of 

 expression caspase 3, 9 and reduced regulatory expression of Bcl-2 proteins and genes (41). Also, in our study, immunohistochemical analysis of Bax and Bcl-2 protein indicated that the expression of Bax protein in the liver of rats receiving NTiO2 was significantly enhanced while the expression of Bcl-2 protein in this group was significantly reduced.

Shukla et al. (2011) reported that NTiO2 induced apoptosis in human HepG2 cell lines in vitro. They reported that administration of very small amounts of NTiO2 (1 μg / ml) resulted in DNA damage and an increase in the expression of apoptosis proteins such as Cyto-c, Bax, P53, Apaf-1, caspase-9, and caspase-3, as well as the reduction of expression of apoptosis-inhibiting proteins such as Bcl-2 (42). Further, Li et al. indicated that NTiO2 anatase at 150 mg / kg led to DNA fragmentation of the rat liver cells and eventually apoptosis in these cells (43). 

In addition to apoptosis, necrosis was found in the liver tissue of animals treated with NTiO2 (44). It was demonstrated that apoptosis and necrosis take place in the cells treated with NTiO2, and the occurrence of a specific cell mortality pattern would be affected by the particles features and the types of cells (45). Further, extensive research has shown that necrosis and apoptosis would not be a fully independent procedure (46), particularly during the apoptosis of several cells (47). According to the IHC and TUNEL outcomes, GA could efficiently reduce the apoptosis and necrosis induced with NTiO2 in the liver. In addition, the hepatocyte apoptotic index was considerably reduced in the NTiO2+GA group in comparison with the NTiO2-intoxicated animals. Another study conducted by Gwak et al. found that GA would attenuate the HMGB1-induced hepatocyte apoptosis (48). 

Based on our outcomes, it was found that GA pretreatment suppressed the Bax protein expression though greater Bcl-2 expression. Tang et al. found that GA mitigated endotoxin-induced liver damage after a relative hepatectomy in the rats, and indicated that it considerably reduced the Caspase 3 expression and release of cytochrome c from mitochondrial to cytoplasm. Further, GA enhanced the expression of nuclear genes related with cell proliferation, suggesting the role of this material in hepatic repair (49). Research results in 2007 suggested that gold nanoparticles in the 2–40 nm interval were taken up primarily by Kupffer cells in the liver and secondarily by macrophages in the spleen and in other places. They stated that endocytosis by macrophages seems not to be the only way that the organism applies to eliminate 2-nanometer gold particles. They hypothesized that part of these tiny nanoparticles was released into the urine as a result of simple in the renal glomeruli (50). Moller et al. found that ultrafine particles cause cytoskeletal toxicity in vitro in macrophages, which can cause cellular dysfunctions, such as impaired proliferation, impaired phagocytic activity, and retarded intracellular transport processes as well as increased cell stiffness, which eventually can result in impaired defense ability in the lungs (51,52). According to our study, it is possible that GA enhances the clearance rate of NTiO2 in the liver via enhancing the phagocytic capability of the Kupffer cells.

Another study by Duan et al. reported that NTiO2 led to apoptosis of the spleen cells and that NTiO2 activated Caspase 3.9, decreased the expression of Bcl-2 proteins and genes, and enhanced the genes expression and proteins of cytochrome C and Bax (53). Studies have also shown that antioxidants such as Green Tea, GSPE, and Resveratrol inhibit apoptosis by controlling the effects of caspases or Bax / Bcl-2 (54).

In conclusion, according to histopathological findings, the present data suggest that GA can effectively protect against NTiO2- induced apoptosis. Nevertheless, these data should not be readily extrapolated to the human population. However, this information does provide a stimulus for true clinical investigations.
